# Inheritance and heritability of deltamethrin resistance under laboratory conditions of *Triatoma infestans* from Bolivia

**DOI:** 10.1186/s13071-015-1211-9

**Published:** 2015-11-16

**Authors:** Marinely Bustamante Gomez, Grasielle D’Avila Caldas Pessoa, Aline Cristine Luiz Rosa, Jorge Espinoza Echeverria, Liléia Gonçalves Diotaiuti

**Affiliations:** Laboratório de Referência em Triatomíneos e Epidemiologia da Doença de Chagas, Centro de Pesquisas René Rachou - FIOCRUZ Minas, Belo Horizonte, Brazil

**Keywords:** *Triatoma infestans*, Inheritance, Heritability, Insecticide resistance, Deltamethrin, Control

## Abstract

**Background:**

Over the last few decades, pyrethroid-resistant in *Triatoma infestans* populations have been reported, mainly on the border between Argentina and Bolivia. Understanding the genetic basis of inheritance mode and heritability of resistance to insecticides under laboratory conditions is crucial for vector management and monitoring of insecticide resistance. Currently, few studies have been performed to characterize the inheritance mode of resistance to pyrethroids in *T. infestans*; for this reason, the present study aims to characterize the inheritance and heritability of deltamethrin resistance in *T. infestans* populations from Bolivia with different toxicological profiles.

**Methods:**

Experimental crosses were performed between a susceptible (S) colony and resistant (R) and reduced susceptibility (RS) colonies in both directions (♀ x ♂ and ♂ x ♀), and inheritance mode was determined based on degree of dominance (DO) and effective dominance (D_*ML*_). In addition, realized heritability (h^2^) was estimated based on a resistant colony, and select pressure was performed for two generations based on the diagnostic dose (10 ng. i. a. /nymph). The F1 progeny of the experimental crosses and the selection were tested by a standard insecticide resistance bioassay.

**Results:**

The result for DO and D_*ML*_ (< 1) indicates that resistance is an incompletely dominant character, and inheritance is autosomal, not sex-linked. The LD_50_ for F1 of ♀S x ♂R and ♂S x ♀R was 0.74 and 3.97, respectively, which is indicative of dilution effect. In the resistant colony, after selection pressure, the value of h^2^ was 0.37; thus, the LD_50_ value increased 2.25-fold (F2) and 26.83-fold (F3) compared with the parental colony.

**Conclusion:**

The inheritance mode of resistance of *T. infestans* to deltamethrin, is autosomal and an incompletely dominant character; this is a previously known process, confirmed in the present study on *T. infestans* populations from Bolivia. The lethal doses (LD_50_) increase from one generation to another rapidly after selection pressure with deltamethrin. This suggests that resistance is an additive and cumulative factor, mainly in highly structured populations with limited dispersal capacity, such as *T. infestans*. This phenomenon was demonstrated for the first time for *T. infestans* in the present study. These results are very important for vector control strategies in problematic areas where high resistance ratios of *T. infestans* have been reported.

## Background

The prevention of Chagas disease is primarily based on vector control, using mainly pyrethroid insecticides [1]. Successful control of *Triatoma infestans* in Brazil, Chile, Uruguay, and drastic reduction of it in large parts of Paraguay and Argentina, are both clearly indicative of its susceptibility to pyrethroid insecticides in its largest area of distribution [2]. By contrast, resistance to pyrethroids in *T. infestans* has been detected since the year 2000 [3] in several areas of Argentina, Bolivia and Paraguay [4–11]. Failure in vector control is considered to be due to low insecticide efficacy when applied to peridomestic structures, unsustainability of vector control interventions, and high levels of insecticide resistance, reported mainly on the border between Argentina and Bolivia, in the biogeographic region of the Gran Chaco [6, 9, 10, 12].

Genetic factors and intensive insecticide application are responsible for the rapid selection of resistance in many insects and mites [13]. Several factors influence the development of insecticide resistance in insect vectors: volume and frequency of insecticide applications (selective pressures), operational failures, and inherent bionomical characteristics of the insect species involved, such as life cycle, abundance, rapid reproduction, and insect migration [14].

Laboratory models are key to understanding the underlying genetics of inheritance and heritability of insecticide resistance. Conclusions drawn from models are fundamental to improve vector management and resistance monitoring [15]. The inheritance mode of pyrethroid resistance was studied in *T. infestans*, and it showed that resistance is autosomal and shows incomplete dominance, involving at least three genes [16, 17]. Therefore, this study aims to characterize inheritance and heritability of insecticide resistance of *T. infestans* populations from Bolivia to deltamethrin with different toxicological profiles.

## Methods

### Insects

We used the F2 generation of three colonies of *T. infestans* from Bolivia, with different toxicological profiles to deltamethrin, previously characterized by Gomez et al. [18]: a susceptible colony (S) from San Silvestre (19°21’21”S/62°34’10”W) Santa Cruz Department; a reduced susceptibility colony (RS) from Rancho Nuevo (19°26’22”S/62°34’05”W) Santa Cruz Department, and a resistant colony (R) from Villa Montes (21°09’02”S/63°21’56”W) Tarija Department. Details of lethal dose 50 (LD_50_) and resistance ratio 50 (RR_50_) are shown in Table 1.

**Table 1:** Toxicological profiles to deltamethrin for colonies of *T. infestans*: *susceptible (S), *reduced susceptibility (RS), *resistant (R) and reciprocally cross progenies: ♀S x ♂RS; ♂S x ♀RS; ♀S x ♂R; ♂S x ♀R

Population	N°	Slope +/− SD	*X* ^*2*^ *(df)*	P	LD_50_ (95 % CI)	RR_50_ (95 % CI)	DO	D_ML_
*S	300	1.97+/− 0.05	0.51 (6)	0.00	0.26 (0.21–0.32)	-	-	-
*RS	390	4.43+/− 0.02	2.41 (10)	0.01	2.09 (1.93–2.27)	5.04		
*R	360	2.25 +/− 0.04	3.05 (9)	0.04	54.23 (45.54–63.32)	129.12		
♀S x ♂RS	210	5.44 +/− 0.76	5.34 (4)	0.23	0.65 (0.55–0.76)	2.64 (2.12–3.29)	−0.08	−1.37
♂S x ♀RS	300	4.33 +/− 0.43	4.30 (7)	0.04	1.30 (1.18–1.43)	5.27 (4.22–6.52)	0.54	−8.07
♀S x ♂R	360	4.44 +/− 0.46	3.18 (7)	0.21	0.74 (0.67–0.81)	2.99 (2.39–3.73)	−0.61	0.00
♂S x ♀R	210	5.86 +/− 0.78	3.17 (6)	0.40	3.97 (3.62–4.30)	16.06 (12.93–19.94)	0.02	0.76

*: Colony characterized by Gomez et al. [18]; N°: number of individuals used; SD: standard deviation; *X* ^*2*^: chi-square test; *df*: degrees of freedom; P: probability value; LD_50_: insecticide dose that killed 50 % of the population [ng/insect); CI: confidence intervals; RR: resistance ratio; ♀: female; ♂: male; DO: Degree of dominance; D_ML_: effective dominance

The calculation of the RR_50_ of the experimental cross was performed with the susceptible colony (S), because during the study carried by Gomez et al. [18], this colony was significantly more susceptible than the reference lineage (SRL) of *T. infestans* from Centro de Investigaciones de Plagas e Insecticidas (CIPEIN) (*X*^2^*p* < 0.05). However, the calculation of the RR_50_ for estimation of heritability was performed with the SRL of *T. infestans*.

### Experimental matings

#### Resistance inheritance (cross)

To study inheritance, male and female fifth-instar nymphs were first identified and maintained individually, until the imaginal moult. Then, triatomine couples were formed, and reciprocal crosses were carried out as follows: ♀S x RS♂; S♂ x ♀RS; ♀S x R♂ and S♂ x ♀R. All couples were maintained in plastic cages (5 cm diameter, 10 cm height), feeding was carried weekly with chicken blood (*Gallus gallus*) (ethical approval of FIOCRUZ No 41/14-2). The F1 progeny was tested through a standard bioassay.

#### Estimation of realized heritability

The evaluation of heritability of resistance was performed with the F1 generation of the resistant colony from Villa Montes Tarija Department (RR_50_ = 129.12), and the individuals selected were those that survived the application of the diagnostic dose of deltamethrin (10 ng a.i./nymph) for successive generations (F2 and F3 survivors); they were then tested by standard bioassays.

### Bioassays

Serial dilutions of technical grade deltamethrin were prepared in acetone. For each concentration, three repetitions were carried out with ten first-instar nymphs, descendant from each of the experimental crosses (the corresponding F generation for each experiment) (five days old, fasting, weight = 1.2 ± 0.2 mg). Diluted insecticide (0.2 μL) was applied on the dorsal abdomen, according to the procedures recommended by the World Health Organization - WHO [19] and Pessoa [20]. For each experimental cross, at least eight doses of insecticide were applied in order to kill between >0 % to <100 % of the individuals (0.35 to 1500.00 ng a. i./nymph). Acetone was applied to the control group. Mortality was assessed 72 h after application, and it was determined by the inability or lack of coordination of the nymphs to move from the center to the edge of the filter paper (7 cm in diameter). Signs of paralysis and lack of response to external stimuli were also taken into consideration. During and after the experiment, the insects were kept under controlled temperature and humidity conditions (25 °C ±1 °C; 60 % ±10 % RH).

### Data analysis

#### Toxicological data

Data from dose response tests from each experiment were analyzed using the PoloPlus software, version 2.0 [21]. Estimations were made of the slope, the lethal doses required to kill 50 % of treated individuals (LD_50_) and confidence intervals (CIs.). The resistance Ratio (RR_50_) was calculated by dividing the LD_50_ value of each experimental cross by the LD_50_ value of the susceptible colony. Parallelism tests were also conducted according to Robertson et al. [22].

#### Degree of dominance

Degree of dominance (DO) for resistance was calculated according to Stone [23] and Preisler et al. [24] using the following formula:

$$ \mathrm{DO}=\frac{2{X}_3-{X}_2-{X}_1}{X_2-{X}_1} $$

Where: X_1_ = log (LD_50_) of the susceptible colony (S); *X*_2_ = log (LD_50_) of the RS or R colony and X_3_ = log (LD_50_) in the reciprocal progeny (F1).

The level of dominance ranges from 0 to 1.0 (i.e. values below 1.0 indicate complete recessiveness and values equal to 1.0 indicate complete dominance).

#### Effective dominance

Effective dominance (*D*_*ML*_) was estimated according to Bourguet et al. [25] and Abbas et al. [26] using the following formula:

$$ {\mathrm{D}}_{ML}=\frac{\mathrm{M}{\mathrm{X}}_3\;\hbox{-}\;\mathrm{M}{\mathrm{X}}_1}{\mathrm{M}{\mathrm{X}}_2\;\hbox{-}\;\mathrm{M}{\mathrm{X}}_1} $$

Where: MX_1_, MX_2_ and MX_3_ represent the mortality percentages for the susceptible, the RS or the R colony and the reciprocal cross progeny (F1), for doses that cause 100 % mortality of the SRL (2.76 ng a. i./nymph treated). D_ML_ expresses effective dominance at a given dose of use, and ranges between 0.0 (survival is recessive) and 1.0 (survival is dominant).

#### Estimation of realized heritability

Following the method of Falconer et al. [27] and Tabashnik [28], realized heritability (h2) of deltamethrin resistance was estimated as follows:

$$ {\mathrm{h}}^2=\frac{\mathrm{R}}{\mathrm{S}} $$

In the above equation, R (selection response) was estimated as follows:

$$ \mathrm{R}=\frac{\mathrm{Lo}{\mathrm{g}}_{\mathrm{final}}\;\mathrm{L}{\mathrm{D}}_{50}-\mathrm{L}\mathrm{o}{\mathrm{g}}_{\mathrm{initial}}\;\mathrm{L}{\mathrm{D}}_{50}}{\mathrm{N}} $$

Final LD_50_ is the LD_50_ of the population after 2 selected generations; initial LD_50_ is the LD_50_ of the field population before selection and N is number of generations selected with deltamethrin.

Whereas, S (selection differential) was calculated as follows:

$$ S=i\;x\upsigma \mathrm{p} $$

Where *i* means intensity of selection calculated according to Falconer et al. [27],

σ*p* means phenotypic standard deviation calculated as follows:

$$ \upsigma \mathrm{p}={\left[\left(\mathrm{initial}\;\mathrm{Slope}+\mathrm{final}\;\mathrm{Slope}\right)\;0.5\right]}^{-1} $$

## Results

### Inheritance of resistance (cross)

The LD_50_ values to deltamethrin of all reciprocal crosses carried out of *T. infestans¸* (♀S x ♂RS; ♂S x ♀RS; ♀S x ♂R and ♂S x ♀R) were significantly different and inferior than their parental RS and R colonies (p < 0.05). This is indicative that deltamethrin resistance in *T. infestans* is inherited autosomal, not sex-linked (Table 1).

The LD_50_ progeny values of ♀S x ♂R and ♂S x ♀R were 0.74 and 3.97, respectively (Table 1), showing that they are 46.11-fold and 8.34-fold less tolerant to deltamethrin than their parental insects of the resistant colony. These results indicate a dilution effect among the resistant and susceptible colonies.

Degree of dominance (DO) values for reciprocal crosses were < 1. This result indicated that the deltamethrin resistance character in *T. infestans* is incompletely dominant. The results of effective dominance (D_*ML*_) (<1) suggested that resistance is a recessive character at the discriminant doses used for deltamethrin (Table 1).

### Estimation of realized heritability

After 2 generations of continuous selection of *T. infestans* with deltamethrin, it was observed that the LD_50_ value increased from 54.23 to 121.93 (F2) and 1455.32 (F3); it is 2.25-fold and 26.83-fold more resistant compared with the parental resistant colony (Table 2). The value calculated for estimation of realized heritability (h^2^) was 0.37 (Table 3). These results indicate increased resistance to deltamethrin from one generation to another, under pressure selection, under laboratory conditions.

**Table 2:** Toxicological profiles to deltamethrin in survivors of *T. infestans* resistant colony (F1 and F2), after application of deltamethrin (10 ng a.i./nymph)

Population cross	N°	Slope +/− SD	*X* ^*2*^ *(df)*	P	LD_50_ (95 % CI)	RR_50_ (95 % CI)
CIPEIN (SRL)*	240	2.83+/−0.04	3.43 (4)	0.51	0.42 (0.35–0.49)	-
Resistant (Parental) Colony*	360	2.25 +/− 0.04	3.05 (9)	0.04	54.23 (45.54–63.32)	129.12 (104.32–166.38)
Surviving F1	210	1.68 +/− 0.42	0.59 (5)	0.01	121.93 (93.65–161.66)	299.23 (222.48–402.47)
Surviving F2	180	1.60 +/− 0.45	0.56 (4)	0.00	1455.32 (1022.71–3914.21)	3571.45 (2099.09–6076.53)

SRL: susceptible reference lineage*: Strain characterized by Gomez et al. [18]; N°: number of individuals used; SD: standard deviation; *X* ^*2*^: chi-square test; *df*: degrees of freedom; P: probability value; LD_50_: insecticide dose that killed 50 % of the population [ng/nymph); CI: confidence intervals; RR: resistance ratio

**Table 3:** Estimate of heritability (h^2^) to deltamethrin resistance in survivors of *T. infestans* after insecticide application under laboratory conditions

N^o^ of generations selected	Estimation of mean selection response per generation			Estimation of mean selection differential per generation					
	Initial log LD_50_	Final log LD_50_	Response to selection (R)	*i*	Initial slope	Final slope	σ*p*	Selection differential (S)	h^2^
2	1.73	3.16	0.72	3.79	2.25	1.60	0.52	1.97	0.37

*i* = intensity of selection [44]; σ*p* = phenotypic variation; h^2^ = Estimation of heritability

## Discussion

For *T. infestans*, few studies have been conducted about resistance inheritance. Cardozo et al. [16] and Germano et al. [17], suggested that deltamethrin resistance of *T. infestans* is autosomal, incompletely dominant, and the maternal effects are null. This fact was confirmed during our study; regardless of the direction where crossing was performed (female or male; susceptible colony with resistant or with reduced susceptibility colony), degree of dominance and effective dominance values were < 1, even lower to 0, indicating incomplete dominance. The LD_50_ values to deltamethrin for reciprocal crosses were significantly different and lower than their parental colonies (*p* < 0.05).

Khan et al. [29] stressed that intense selection pressure leads to an increase in resistant genotypes in the population due to the removal of susceptible individuals, increasing the frequency of resistant individuals in a population. However, epistatic interactions can occur, mainly in populations with inbreeding, when each allele contributes to resistance, and the introduction of a susceptible allele dilutes the effect under laboratory conditions [30]. Thus, during our study, the LD_50_ values of experimental crosses among resistant and susceptible colonies were 0.73 (R♀ x S♂) and 3.98 (S♀ x R♂) (Table 1), showing a 46.11-fold and an 8.34-fold decrease in the LD_50_ values when compared with resistant parental insects (LD_50_ = 50.23). This suggests that there is a dilution effect of the resistant character to deltamethrin under laboratory conditions.

Several studies demonstrated high levels of structuring of *T. infestans* populations [31–35, 36] and indicated limited capacity of active dispersal and restricted interbreeding. Germano et al. [37] suggested that geographical structure is present at the microgeographic level, and demonstrated that *T. infestans* populations in different dwellings in the same area have different toxicological profiles to deltamethrin. Selection pressure for insecticide application has a greater effect in populations with limited capacity of dispersal, as is the case of *T. infestans*, than on populations with high dispersal ability. The persistent bug populations that survived insecticide application at local spatial scales support two distinct, but equally worrying scenarios. In the first scenario, operational failures may hinder the control of Triatomine populations, as a result of low quality of the spraying technique and/or low efficacy of pyrethroids, especially in peridomestic structures. In the second scenario, difficulty in control may be due to the intrinsic features of Triatomine bugs, which make them resistant to chemical agents. [32, 38–40].

Different toxicological profiles and mechanisms of resistance were reported for *T. infestans* [7, 8, 11, 41]. Mutation of two different alleles of knockdown resistance (*kdr*) were reported from different populations in the Gran Chaco region in Argentina [7, 41], spanning a different geographical distribution. Coincidently, the resistant population used for the experiment of heritability was collected in the Gran Chaco region, where it can be seen that the LD_50_ value increased from 54.23 (parental) to 121.93 (F2) and 1455.32 (F3) (Table 2). These results suggest that resistance to deltamethrin in *T. infestans* could increase rapidly. Despite their long life cycle, only two generations of continuous laboratory selection were required to increase the level of resistance 26.83-fold. The rapid selection and high levels of resistance in this population are likely to be caused by *kdr* mutation.

Estimation of heritability (h^2^) is a proportion of phenotypic variation accounted by additive genetic variation, which may decrease either due to a decrease in additive genetic variance or to an increase in environmental variance [27]. During our study, the value for estimation of heritability (h^2^) to deltamethrin was 0.37 (Table 3). This lower value was most likely caused by lower phenotypic variation, mainly environmental variation, which indicates the presence of resistant alleles in the study colony [42]. Similarly, the selection was done under laboratory conditions; it does not completely reflect the development of resistance in the field population because of homogeneous environmental conditions [28]. In addition, polygenic and monogenic resistance may occur naturally in insect populations [29]. However, monogenic resistance is less likely to occur under laboratory selection, given the absence of rare variants that may be present in natural insect populations [43, 44].

The laboratory experiments and the use of only one insecticide (deltamethrin) impose limitations compared to field conditions. However, most studies evaluating the susceptibility of insects in the laboratory used deltamethrin (technical grade) as a standard insecticide, because this molecule is stable and there is a great amount of knowledge available about it. As the results in this study only used deltamethrin, it could be reasonably extrapolated to other pyrethroids, but not to other active ingredients (i.e. carbamates).

Moreover, the estimated heritability values provide evidence for the potential of resistance development in the future [15, 29, 30, 43]. Thus, the results of estimation of realized heritability allow the prediction of the number of generations required for the population to increase the level of resistance [28]. It should be noted that this is the first study on estimation of realized heritability in *T. infestans* to deltamethrin, and despite the long life cycle (around 6 months for adults), the values of LD_50_ could be increased considerably (26.83-fold) within two generations. Characterizing the mode of inheritance and determining the estimation of the h^2^ value is an essential tool for assessing sustainability of insecticides on the pest population and vectors of public health importance for resistance management [45]. Therefore, more long-term studies should be conducted to clarify the selection process in bugs, because it is very important for control vector management.

## Conclusion

The results obtained in our study indicate that (1) the inheritance mode of deltamethrin resistance of *Triatoma infestans*, under laboratory conditions, is autosomal and an incompletely dominant character. This is a previously known process, and it was confirmed in other *T. infestans* populations from Bolivia in this study; (2) the lethal doses (LD_50_) and resistance ratio increase from one generation to another rapidly, after selection pressure with deltamethrin. This suggests that resistance is an additive and cumulative factor, mainly in highly structured populations with limited dispersal capacity, such as *T. infestans*. This phenomenon was demonstrated for the first time for *T. infestans* under laboratory conditions in the present study. These results are very important for vector control programs in problematic areas where high resistance ratios of *T. infestans* have been reported.

## Abbreviations

°C: Celsius degree
♀: Female
♂: Male
a.i: Active ingredient
i.e.: For example
mg: Milligrams
ng: Nano grams
RH: Humidity relative
μL: Microliters

## References

[1] Zerba EN (1999). Susceptibility and resistance to insecticides of Chagas disease vectors. Med (B Aires).

[2] Panzera F, Ferreiro MJ, Pita S, Calleros L, Pérez R, Basmadjián Y (2014). Evolutionary and dispersal history of *Triatoma infestans*, main vector of Chagas disease, by chromosomal markers. Infect Genet Evol.

[3] Vassena C, Picollo M, Zerba E (2000). Insecticide resistance in Brazilian *Triatoma infestans* and Venezuelan *Rhodnius prolixus*. Med Vet Entomol.

[4] Alarico AG, Romero N, Hernández L, Catalá S, Gorla D (2010). Residual effect of a micro-encapsulated formulation of organophosphates and piriproxifen on the mortality of deltamethrin resistant *Triatoma infestans* populations in rural houses of the Bolivian Chaco region. Mem Inst Oswaldo Cruz.

[5] Vassena CV, Picollo MI, Santo-Orihuela P, Zerba EN. Desarrollo y manejo de la resistencia a insecticidas piretroides en *Triatoma infestans*: Situación en Bolivia. Cortez MR, Triatominos de Bolivia, Ministerio de Salud y Deportes de Bolivia – Programa Nacional de Chagas, Bolivia 2007, 229–255.

[6] Depickère S, Buitrago R, Siñani E, Baune M, Monje M, Lopez R (2012). Susceptibility and resistance to deltamethrin of wild and domestic populations of *Triatoma infestans* (Reduviidae: Triatominae) in Bolivia: new discoveries. Mem Inst Oswaldo Cruz.

[7] Fabro J, Sterkel M, Capriotti N, Mougabure-Cueto G, Germano M, Rivera-Pomar R (2012). Identification of a point mutation associated with pyrethroid resistance in the para-type sodium channel of *Triatoma infestans*, a vector of Chagas’ disease. Infect Genet Evol.

[8] Germano M, Santo-Orihuela P, Roca-Acevedo G, Toloza A, Vassena C, Picollo M (2012). Scientific evidence of three different insecticide-resistant profiles in *Triatoma infestans* (Hemiptera: Reduviidae) populations from Argentina and Bolivia. J Med Entomo.

[9] Lardeux F, Depickère S, Duchon S, Chavez T (2010). Insecticide resistance of *Triatoma infestans* (Hemiptera, Reduviidae) vector of Chagas disease in Bolivia. Trop Med Int Health.

[10] Picollo MI, Vassena C, Santo Orihuela P, Barrios S, Zaidemberg M, Zerba E (2005). High resistance to pyrethroid insecticides associated with ineffective field treatments in *Triatoma infestans* (Hemiptera: Reduviidae) from Northern Argentina. J Med Entomo.

[11] Santo Orihuela PL, Vassena CV, Zerba EN, Picollo MI (2008). Relative contribution of monooxygenase and esterase to pyrethroid resistance in *Triatoma infestans* (Hemiptera: Reduviidae) from Argentina and Bolivia. J Med Entomo.

[12] Germano M, Acevedo GR, Cueto GM, Toloza A, Vassena C, Picollo M (2010). New findings of insecticide resistance in *Triatoma infestans* (Heteroptera: Reduviidae) from the Gran Chaco. J Med Entomo.

[13] Insecticide Resistance Action Committee: Prevention and Management of Insecticide Resistance in Vectors of Public Health Importance Second Edition, 2011. http://www.irac-online.org/content/uploads/VM-Layout-v2.6_LR.pdf

[14] Hemingway J, Ranson H (2000). Insecticide resistance in insect vectors of human disease. Ann Rev Entomol.

[15] Sayyed AH, Haward R, Herrero S, Ferré J, Wright DJ (2000). Genetic and biochemical approach for characterization of resistance to *Bacillus thuringiensis* toxin Cry1Ac in a field population of the diamondback moth, Plutella xylostella. Appl Environ Microbiol.

[16] Cardozo R, Panzera F, Gentile A, Segura M, Pérez R, Díaz R (2010). Inheritance of resistance to pyrethroids in *Triatoma infestans*, the main Chagas disease vector in South America. Infect Genet Evol.

[17] Germano MD, Vassena CV, Picollo MI (2010). Autosomal inheritance of deltamethrin resistance in field populations of *Triatoma infestans* (Heteroptera: Reduviidae) from Argentina. Pest Manag Sci.

[18] Gomez MB, Pessoa GC, García ALO, Cortez MR, Rosa ACL, Noireau F (2014). Susceptibility to deltamethrin of wild and domestic populations of *Triatoma infestans* of the Gran Chaco and the Inter-Andean Valleys of Bolivia. Parasit Vectors.

[19] World Health Organization (1994). Protocolo de evaluación de efectoinsecticidasobretriatominos. Acta Toxicol.

[20] Pessoa GC, Pinheiro LC (2015). FerrazML, de Mello BV, Diotaiuti L: Standardization of laboratory bioassays for the study of *Triatoma sordida* susceptibility to pyrethroid insecticides. Parasit Vectors.

[21] LeOra Software. PoloPlus user's manual, version 2.0; 2005.

[22] Robertson JL, Russell RM, Preisler HK, Savin N (2007). Bioassays with arthropods.

[23] Stone BF (1968). A formula for determining degree of dominance in cases of monofactorial inheritance of resistance to chemicals. Bull World Health Organ.

[24] Preisler HK, Hoy MA, Robertson JL (1990). Statistical analysis of modes of inheritance for pesticide resistance. J Econ Entomo.

[25] Bourguet D, Genissel A, Raymond M (2000). Insecticide resistance and dominance levels. J Econ Entomo.

[26] Abbas N, Khan HAA, Shad SA (2014). Cross-resistance, genetics, and realized heritability of resistance to fipronil in the house fly, *Musca domestica* (Diptera: Muscidae): a potential vector for disease transmission. Parasitol Res.

[27] Falconer DS, Mackay TF (1996). Introduction to quantitative genetics 4th ed. Trends Genet.

[28] Tabashnik BE (1991). Determining the mode of inheritance of pesticide resistance with backcross experiments. J Econ Entomo.

[29] Khan H, Abbas N, Shad SA, Afzal MBS (2014). Genetics and realized heritability of resistance to imidacloprid in a poultry population of house fly, *Musca domestica L*. (Diptera: Muscidae) from Pakistan. Pestic Biochem Physiol.

[30] Prasad T, Shetty N (2013). Autosomal inheritance of alphamethrin, a synthetic pyrethroid, resistance in *Anopheles stephensi–Liston*, a malaria mosquito. Bull Entomo Res.

[31] Perez De Rosas AR, Segura EL, Fichera L, García BA (2008). Macrogeographic and microgeographic genetic structure of the Chagas’ disease vector *Triatoma infestans* (Hemiptera: Reduviidae) from Catamarca. Argentina Genetica.

[32] Gaspe MS, Gurevitz JM, Gürtler RE, Dujardin JP (2013). Origins of house reinfestation with *Triatoma infestans* after insecticide spraying in the Argentine Chaco using wing geometric morphometry. Infect Genet Evol.

[33] Marcet P, Mora M, Cutrera A, Jones L, Gürtler R, Kitron U (2008). Genetic structure of *Triatoma infestans* populations in rural communities of Santiago del Estero, northern Argentina. Infect Genet Evol.

[34] Perez De Rosas AR, Segura EL, Garcia BA (2007). Microsatellite analysis of genetic structure in natural *Triatoma infestans* (Hemiptera: Reduviidae) populations from Argentina: its implication in assessing the effectiveness of Chagas’ disease vector control programmes. Mol Ecol.

[35] Pizarro JC, Gilligan LM, Stevens L (2008). Microsatellites reveal a high population structure in *Triatoma infestans* from Chuquisaca. Bolivia PLoS Negl Trop Dis.

[36] Quisberth S, Waleckx E, Monje M, Chang B, Noireau F, Brenière SF (2011). “Andean” and “non-Andean” ITS-2 and mtCytB haplotypes of *Triatoma infestans* are observed in the Gran Chaco (Bolivia): population genetics and the origin of reinfestation. Infect Genet Evol.

[37] Germano MD, Picollo MI, Mougabure-Cueto GA (2013). Microgeographical study of insecticide resistance in *Triatoma infestans* from Argentina. Acta Trop.

[38] Cecere MC, Vazquez-Prokopec GM, Ceballos LA, Boragno S, Zárate JE, Kitron U (2013). Improved chemical control of Chagas disease vectors in the dry Chaco region. J Med Entomol.

[39] Gurevitz JM, Gaspe MS, Enríquez GF, Vassena CV, Alvarado-Otegui JA, Provecho YM, et al. Unexpected failures to control Chagas disease vectors with pyrethroid spraying in northern Argentina. J Med Entomol. 2012;49(6):1379–86. http://dx.doi.org/10.1603/ME11157 10.1603/me11157 PMC3760256 23270166

[40] Organización Panamericana de Salud: II Reunión Técnica Latinoamericana de Monitoreo de Resistencia a Insecticidas en Triatomineos Vectores de Chagas Panamá. 11 al 13 de Abril 2005.

[41] Capriotti N, Mougabure-Cueto G, Rivera-Pomar R, Ons S (2014). L925I Mutation in the Para-Type Sodium Channel Is Associated with Pyrethroid Resistance in *Triatoma infestans* from the Gran Chaco Region. PLoS Negl Trop Dis.

[42] Zhang L, Shi J, Gao X (2008). Inheritance of beta‐cypermethrin resistance in the housefly *Musca domestica* (Diptera: Muscidae). Pest Manag Sci.

[43] Yuan JS, Tranel PJ, Stewart CN (2007). Non-target-site herbicide resistance: a family business. Trends Plant Sci.

[44] McKenzie JA, Parker AG, Yen JL (1992). Polygenic and single gene responses to selection for resistance to diazinon in *Lucilia cuprina*. Genetics.

[45] Sayyed AH, Attique MNR, Khaliq A, Wright DJ (2005). Inheritance of resistance and cross‐resistance to deltamethrin in *Plutella xylostella* (Lepidoptera: Plutellidae) from Pakistan. Pest Manag Sci.

